# Measuring what matters – information systems for management of chronic disease in primary healthcare settings in low and middle-income countries: challenges and opportunities

**DOI:** 10.1017/S204579602000030X

**Published:** 2020-05-11

**Authors:** R. Mayston, K. Ebhohimen, K. Jacob

**Affiliations:** 1 King's Global Health Institute, King's College London, Franklin Wilkins Building, Stamford Street, London, SE1 9NH, UK; 2 MindWave Ventures, 4a White Horse Mews, 37 Westminster Bridge Rd, London, SE1 7QD, UK

**Keywords:** Chronic care, community mental health, health service research, multimorbidity, quality of care

## Abstract

Effective health information systems are essential to the delivery of high-quality community-based care for chronic disease which will be needed to address the changing healthcare needs of populations in low and middle-income country settings. Health management information systems (health service data collected at facility level) and electronic health records (data organised by individual patients) may support the measurement-based, collaborative approach that is central to the chronic care model, which has been adopted as the basis for task-shared models of care for mental health and non-communicable disease. We used the performance of routine information systems management to guide our commentary on the evidence-base about information systems to support chronic care. We found that, despite an appetite for using the information to support decision-making around service planning, this rarely happens in practice, reasons include that data is not perceived to be of good quality or fit for purpose. There is often a mismatch between technology design and the availability of specialised knowledge and infrastructure. However, when data collection is designed in collaboration with local stakeholders, there is some evidence of success, demonstrated by completion and accuracy of data forms. Whilst there are global targets for the development of health information systems and progress on these is undoubtedly being made, indicators for chronic disease are seldom prioritised by national governments and there is insufficient decentralisation to facilitate local data-driven decision-making. Our recommendations for future research and development, therefore, focus upon the need to integrate context into the design of information systems: through building strong multisectoral partnerships, ensuring newly developed indicators are well aligned to service models and using technology that is a good fit with local infrastructure. This approach will be necessary if information systems are to deliver on their potential to drive improvements in care for chronic disease.

Health information systems (HIS) have been described as ‘a set of components and procedures, organised with the objectives of generating information, which will improve health care management decisions at all levels of the health system’ (Lippeveld *et al*., 2000). Designed to capture, organise and aggregate data that can be used to manage individual patient care, support quality improvement activities and allocation of resources from facility to national level; HIS have been identified as a key foundation of a high-quality health system by the recent Lancet Commission (Kruk *et al*., 2018). In theory, HIS help to direct scarce resources to those most in need, extending health systems' reach to support currently underserved groups including people living with poor mental health, non-communicable diseases (NCDs) and multimorbidity (Bauer *et al*., 2014). Given that a community-based chronic care model is optimal for effective management of these conditions, the improvement of information systems in primary healthcare (PHC) is crucial (Wagner *et al*., 1996; van Ginneken *et al*., 2011; WHO 2016).

There are three core motivations for prioritising the development of high-quality HIS within PHC in lower and middle-income countries (LMIC) as an important strategy for health systems strengthening. First, sustainable development goal (SDG)-3 puts the health system, its potential for positive impacts upon population health and promotion of equity, at the centre of global health and development agendas. There is both a renewed focus upon defining high-quality health systems and increased scrutiny of key deficits of current systems (Kruk *et al*., 2018). Second, changing needs of populations, driven by demographic and epidemiological transformations, such as, population ageing and multimorbidity have resulted in a renewed focus on the importance of primary care, as well as a shift towards more value driven, person-centred care which attempts to address social determinants, prevention and case management for people living with multiple conditions and complex symptoms (Fairall and Bateman, 2017). Although the SDGs make no explicit mention of PHC, it is implicit to many of the goals, with the centrality of PHC to achieving broader development aims reinforced in the Alma Ata declaration (Jordans *et al*., 2019). Last, researchers have identified significant treatment gaps for chronic illnesses, particularly in LMIC settings where health systems are fragmented, and funding is scarce (Mukeshimana and McHunu, 2017). Gaps exist across the continuum of care, from detection to effective management (Lupafya *et al*., 2016; Fekadu *et al*., 2017; Roberts *et al*., 2018; Qarni *et al*., 2019; Werfalli *et al*., 2019). Policies, plans and services are being rapidly developed to integrate mental healthcare and management of NCDs into some of the world's most poorly resourced health systems, which were originally designed to address acute illness episodes due to infectious disease (Mugisha *et al*., 2017). These systems now need to adapt rapidly to meet the needs for chronic care among people living with long-term illness.

In this commentary, we explore the potential role of information systems in health systems strengthening to support effective treatment of mental health problems in primary care. First, we describe the information needs of chronic care, define HIS and describe its core components (health management information systems (HMIS) and electronic health records (EHRs)). Second, with reference to the performance of routine information systems management (PRISM) framework for the evaluation for HIS (Aqil *et al*., 2009), we describe current challenges and opportunities to the introduction of HIS to support chronic care in PHC in LMIC. Last, we make recommendations for future directions for research and development in this area.

## HIS: HMIS, EHRs and the chronic care model

As an important component of the HIS, HMIS refer to health services data collected at a facility level, which can include characteristics such as systems data (personnel, training); activities (types of intervention delivered); service-user data (diagnoses, socio-demographic data, co-morbidities) (Lora *et al*., 2017). A key purpose of this aggregated data is to support short-term management, service planning and resource prioritisation. In the long-term, HMIS may facilitate better governance, transparency, evidence-based decision-making; and quality and performance-based financing, ultimately supporting the health system to better meet the needs of the population (Upadhaya *et al*., 2016). EHRs, differ from HMIS in that they are organised by individual patients rather than facilities, in order to facilitate tracking of service-users wherever they are present, supporting the sharing of information between points of care and the use of this data to guide clinical decision-making, thereby driving improvements in continuity and overall quality of care (Katurura and Cilliers, 2018).

Chronic care relies upon a measurement-based approach with the sharing of information between team members (Wagner *et al*., 1996). This model has been adapted for use in low resource settings where ‘task-shared’ models of mental healthcare have been widely researched and integrated into national policies and plans which are now beginning to be implemented at scale. Task-shared teams for mental healthcare typically include community health workers who visit households in their communities, medical officers or primary healthcare nurses and minimal input from specialists (van Ginneken *et al*., 2011). For mental health in LMIC, often community health workers will identify people who may require treatment before screening/diagnosis takes place at a primary healthcare centre. Depending on severity and treatment options available, formal diagnosis and initiation of treatment may take place at a secondary/tertiary facility. Increasingly, the staff at PHCs are trained to deliver psychosocial interventions for mental health, in line with WHO's Mental Health Global Action Plan (mhGAP) recommendations (WHO 2016). The development of structured care pathways, whereby care is organised according to explicit goals, roles and care processes and outcomes are documented and are likely to be essential to the success of these interventions (Schrijvers *et al*., 2012). Currently, in many settings, little information is recorded about mental health or NCD service-users in primary care in LMIC, other than perhaps their attendance and diagnosis, often recorded using paper registers, making high quality, longitudinal, patient-centred care impossible.

HIS may be harnessed to meet the needs of chronic care models for mental health in PHCs of LMICs (Young *et al*., 2007). Some examples can be seen from studies which have used HIS to deliver better management of diabetes (Young *et al*., 2004) and schizophrenia [19] in HIC settings. These studies have shown that HIS can effectively enable: (a) clinicians in clinical decision-making based on accessible clinical information about their individual patients, (b) clinical managers in the identification of pervasive problems in care, monitoring of performance, and improvement access to necessary treatments, and (c) public health administrators in understanding disease burden, monitoring quality and population health outcomes, and in creating and coordination of patient-centred community-based care.

## Challenges and opportunities

Challenges of and opportunities for effectively using information systems for chronic care of mental health in PHCs of LMICs can be analysed in terms of behavioural, technical and organisational determinants of HIS performance. This approach is based on the PRISM framework for the evaluation for HIS (Aqil *et al*., 2009). Consideration of behavioural determinants covers all aspects of human involvement in the performance of information systems. This relates to ‘how people react to and use information’ (Aqil *et al*., 2009). In EMERALD, a recent multi-country study, healthcare workers in Uganda and Nepal requested incentives to support their new role for collecting mental health data; whilst in the three other study countries (Ethiopia, Nigeria, South Africa), there was no evidence to suggest that data was being used to support service improvement (Ahuja *et al*., 2019). Other authors have suggested that, in general, data from HMIS is not commonly used for the district or community-level planning or resource allocation (Wickremasinghe *et al*., 2016) and that data derived from HMIS are perceived to be poor quality and unreliable (Nnaji *et al*., 2010). There is insufficient management and administrative capacity to use data effectively to support decision-making (Mubyazi *et al*., 2004), and a lack of healthcare personnel with health informatics training to develop and maintain systems (Katurura and Cilliers, 2018). However, there is research evidence to suggest that there is an appetite for using data to support care delivery and planning: a qualitative evaluation suggested that healthcare workers and managers recognised the usefulness of collecting and aggregating data on mental health, in the same study, in Nigeria and Uganda, managers have expressed enthusiasm about using data to support service planning (Ahuja *et al*., 2019).

From the technical determinants perspective, there is a need to consider factors that are related to the specialised know-how and technology which is necessary to develop, manage and improve HIS processes and performance (Aqil *et al*., 2009). There has been a proliferation of potential technological solutions, though there remains a high failure with the extent of adoption (Yogeswaran and Wright, 2010; Kiberu *et al*., 2017). It has been observed that introducing EHR in the context of fragmented and under-resourced health systems can be a threat to feasibility as well as perceived value. There are deficits in IT infrastructure, including, lack of computers, networking equipment, internet connectivity (Akhlaq *et al*., 2016; Katurura and Cilliers, 2018). In addition, interoperability between different EHR systems is poor and data related to population mental health and NCDs are particularly weak, with indicators seldom included in national information systems, offering a description of population burden and needs that is incomplete and unreliable at best (Jordans *et al*., 2019). When collection and aggregation of data on mental health and NCDs are introduced, there are often concerns about additional time taken and the challenge of integration of the new data collection with existing information systems (Jordans *et al*., 2019). On the other hand, publications from EMERALD demonstrate the feasibility of the development and implementation of mental health indicators designed to target universal health coverage in India, Nepal, Ethiopia, Nigeria, South Africa and Uganda (Upadhaya *et al*., 2016). Forms were locally developed and used to capture the following indicators: service utilisation by disorder, severity, functioning, administered treatment, referral, follow-up and payment for services (Jordans *et al*., 2019). High levels of completeness and correctness of data were achieved at time points measured, indicating overall feasibility and acceptability (Jordans *et al*., 2019).

Finally, the organisational determinants perspective considers the contextual factors which influence performance through organisational rules, values and practices (Aqil *et al*., 2009). Few LMIC governments prioritise the collection of mental health indicators as part of routine HMIS. Where data is gathered, these are often designed to be collected for the purposes of monitoring and upward reporting, limiting its capacity to support local decision-making (Mubyazi *et al*., 2004). There is often insufficient decentralisation to enable local planners to make meaningful decisions and a lack of co-ordination between different government policies and departments (Bossert and Beauvais, 2002). Changes to government and political unrest commonly disrupt implementation. Nonetheless, there are signs of positive developments in policy development and implementation. The Lancet Commission found that in 2015, 34 LMICs had adopted national EHRs systems and 41 LMICs used District Health Information Software (DHIS) 2 at a national scale for aggregate reporting from electronic or paper registers in facilities (Kruk *et al*., 2018). One of the four objectives of the World Health Organization's (WHO) Mental Health Action Plan for 2013–2020 focuses on ‘strengthening information systems, evidence and research for mental health’ with a related target which aims for 80% of member states to report core mental health indicators through their routine monitoring systems by 2020 (WHO, 2013). To guide the process of strengthening mental health information systems (MHIS), the WHO developed a module for MHIS in its Mental Health Policy and Service Guidance Package (World Health Organization, 2005). The MHIS module proposes evidence-based principles and steps for MHIS development. In LMICs, there are several examples of EHRs for particular groups of patients, often those living with HIV (Manders *et al*., 2010; Waters *et al*., 2010). A number of LMICs are in the process of developing or introducing national systems of EHRs (e.g. India, South Africa, China, Ethiopia, Pakistan and Thailand) (Akhlaq *et al*., 2016).

Work within the ASSET programme https://healthasset.org/ Global Health Research Unit on Health System Strengthening in Sub-Saharan Africa (2017–2021, NIHR grant number: GHRU 16/136/54) aims to address some of the current evidence gaps for HMIS to support primary mental healthcare by drawing upon principles of user-led design to develop an application to support primary healthcare for depression and co-morbid chronic physical illness. Working in rural Ethiopia, the ASSET programme will explore the practicability of phones, linked to cloud servers to collect, aggregate, analyse and use HMIS data which consists of five indicators: (1) detection of a condition, (2) engagement on a care pathway, (3) intervention adherence, (4) retention and (5) ‘treatment to target’. On detection, the application will generate a care pathway, with prompted assessments and associated actions at each follow-up appointment. Patient data will be accessed by healthcare workers providing care in order to support continuity of care. Escalating reminders of overdue tasks and follow-up from supervisors were found to be effective in reducing delayed actions among community health workers in Tanzania. Within the ASSET application, the patient registry allows healthcare workers to focus attention on patients most in need: a ‘dashboard’, listing all NCD patients within a facility will highlight those who have ‘red flags’ – missed appointments, severe or deteriorating symptoms. Aggregated data can be used to compare the care quality and outcomes for healthcare workers, facilities and districts to; target supervision and support; identify best performing healthcare workers and facilities to mentor others; inform quality improvement initiatives, with real-time data to track effectiveness. We are working with facility managers and quality improvement/information teams to ensure data from the application is useable and attuned to existing reporting and review processes and timelines. A prototype has been built and one round of user testing completed, with a second round planned for 2020.

## Recommendations for future research and development of information systems to support chronic care

1. *Multisectoral collaboration and strong partnerships are essential*: Although more challenging, a systems-thinking approach, involving the full range of stakeholders, is likely to be necessary. Like all health systems strengthening efforts, building strong partnerships will be essential to achieving successful work (Breuer *et al*., 2019). It is likely that in addition to funding secured from donors and research funding, the government will need to allocate resources to secure the sustainability of information systems. Congruence of initiatives with broader policy environments and information systems for other diseases will, therefore, be crucial to success: for researchers, building trusting relationships with Ministries of Health will, therefore, be essential. Capacity-building work with policymakers is likely to be important in increasing understanding of health systems strengthening interventions and research methodologies (Semrau *et al*., 2018). Information systems themselves will need to be well-aligned to both people, service models and accompanying workflows. The organisation of chronic care, in the form of care pathways, identifying different activities, decision points, where these occur, who is involved and how the different activities and actors relate to one another is a vital first step to making a task-shared service work. Research and development work will require collaboration between actors from different disciplines (learning technologists, software designers, clinicians, epidemiologists, service-user researchers), preferably taking an interdisciplinary approach. Participatory research methods will need to be used, both to understand more about individual needs and how information gathering, recording and use might fit into workflows, as well as building understanding and ownership among stakeholders of the information system that emerges from formative work. Building capacity for development and maintenance of HIS, the dissemination and utilisation of data will be a function of strong partnerships as well as being crucial to the success of the system as a whole (Kiberu *et al*., 2017). This is likely to include the development of in-service training modules, mentorship programmes for different cadres of the health system and the development of career pathways for informaticians.

2. *Locally meaningful mental health indicators, aligned to service models must be introduced*: Evidence from the USA and elsewhere suggests that chronic care models supported by appropriate information systems have the potential to help drive quality improvement and support patient-centred care. However, for this to be the case, data collected must be accessible and perceived to be of value to users; users should have sufficient autonomy to make data-informed decisions. The EMERALD programme has demonstrated that it is feasible to develop mental health indicators for primary mental healthcare in LMIC settings, using consensus-building methodologies (Jordans *et al*., 2019). National policymakers and healthcare workers have been the main focus HMIS development work in LMIC. Increasing the involvement of service-users in the development of indicators and information systems will help to improve the accountability of services and the health system as a whole. Likewise, ensuring that systems are in place to facilitate timely access to local planners and managers to data that is of high quality will be essential to initiating quality improvement activities. Incentivisation may be counterproductive. Social determinants of health which are likely to mediate both the severity of mental illness and the effectiveness of interventions may be largely outside of the control of the provider will interact with measures of quality of care, potentially increasing the risk of ‘cherry picking’ of less severely affected service-users in contexts where quality indicators are incentivised (Kilbourne *et al*., 2018). There is limited evidence from TB care that incentivisation of complex objectives that require multiple behaviours may have adverse effects upon outcomes.

3.*Technology used for data collection and management should be appropriate*: To avoid failure, creative approaches are needed to work within the constraints of available infrastructure and technology. This is likely to involve ‘leapfrogging’ of intermediate phases of development used elsewhere, in favour of newer forms of technology which have the potential to contribute to reducing inequities of access to knowledge, information and power more rapidly (Akhlaq *et al*., 2016) (e.g. Mittal *et al*., 2010). For example, researchers have shown that it is feasible and acceptable to introduce smartphones to collect maternal health data in rural Ethiopia (Little *et al*., 2013; Medhanyie *et al*., 2015). Technology choices must be compatible with the local context, for example, having offline functionality in environments where internet connectivity is unreliable and compliance with regulatory and legal frameworks (e.g. Uganda's National eHealth Policy) (Government of Uganda, 2016). Evolving initiatives should therefore be consistent with national government plans, or, at the very least, research teams and donors should ensure that systems have good interoperability, for example, by adhering to specifications for standardised content and structure of core information, such as those set out by Fast Healthcare Interoperability Resources (Braunstein, 2018).

## Conclusions

Adaptation of health systems to ensure they drive the development of high-quality chronic care for mental health and NCDs is essential to meet changing population healthcare needs and global development goals. Effective information systems are crucial to delivering a continuum of care for people living with chronic conditions. Whilst there has been a proliferation of evidence about technology in healthcare, there is a lack of evidence focussed on supporting staff to organise and deliver chronic care. Effective solutions will be those that use technology that feels familiar to users, with a purpose that is clearly defined and understood and that is perceived to have direct utility to multiple uses.
